# Supplementary material for: Is the Glycoprotein Responsible for the Differences in Dispersal Rates between Lettuce Necrotic Yellows Virus Subgroups?
Source: Viruses. 2022 Jul 20;14(7):1574. doi: 10.3390/v14071574 (PMC9316239; doi:10.3390/v14071574)
Supplement: Supplementary file 1 [file viruses-14-01574-s001.zip › G Protein manuscript supplementary figures.pptx]

## Slide 1
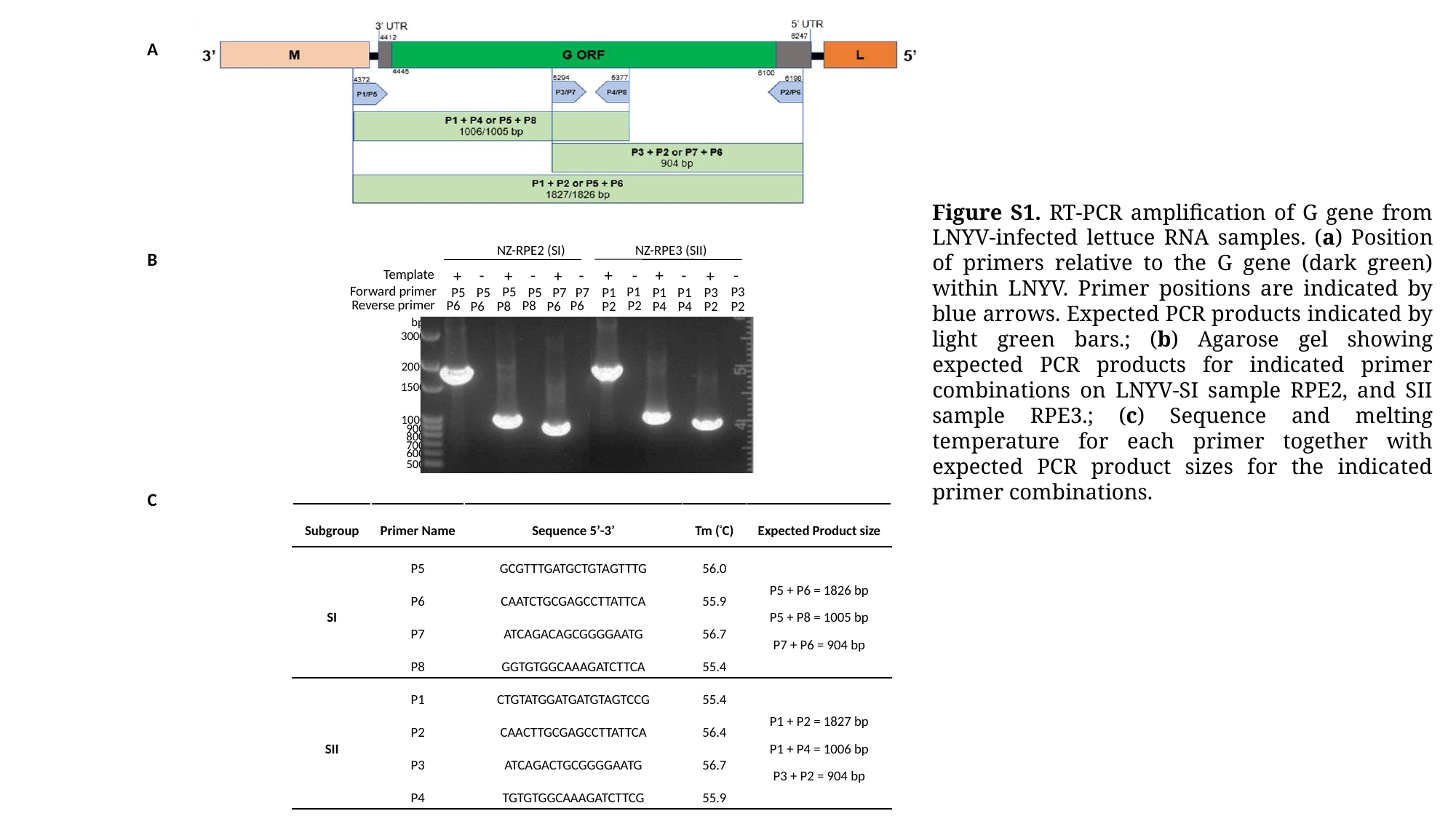

A
Figure S1. RT-PCR amplification of G gene from LNYV-infected lettuce RNA samples. (a) Position of primers relative to the G gene (dark green) within LNYV. Primer positions are indicated by blue arrows. Expected PCR products indicated by light green bars.; (b) Agarose gel showing expected PCR products for indicated primer combinations on LNYV-SI sample RPE2, and SII sample RPE3.; (c) Sequence and melting temperature for each primer together with expected PCR product sizes for the indicated primer combinations.
NZ-RPE2 (SI)
NZ-RPE3 (SII)
-
-
-
+
+
+
P3
P1
P1
P3
P1
P1
P2
P4
P2
P2
P2
P4
-
-
-
+
+
+
Template
Forward primer
P5
P7
P5
P5
P7
P5
Reverse primer
P8
P6
P6
P6
P8
P6
bp
3000
2000
1500
1000
900
800
700
600
500
B
C
| Subgroup | Primer Name | Sequence 5’-3’ | Tm (°C) | Expected Product size |
| --- | --- | --- | --- | --- |
| SI | P5 | GCGTTTGATGCTGTAGTTTG | 56.0 | P5 + P6 = 1826 bp P5 + P8 = 1005 bp P7 + P6 = 904 bp |
| | P6 | CAATCTGCGAGCCTTATTCA | 55.9 | |
| | P7 | ATCAGACAGCGGGGAATG | 56.7 | |
| | P8 | GGTGTGGCAAAGATCTTCA | 55.4 | |
| SII | P1 | CTGTATGGATGATGTAGTCCG | 55.4 | P1 + P2 = 1827 bp P1 + P4 = 1006 bp P3 + P2 = 904 bp |
| | P2 | CAACTTGCGAGCCTTATTCA | 56.4 | |
| | P3 | ATCAGACTGCGGGGAATG | 56.7 | |
| | P4 | TGTGTGGCAAAGATCTTCG | 55.9 | |

## Slide 2
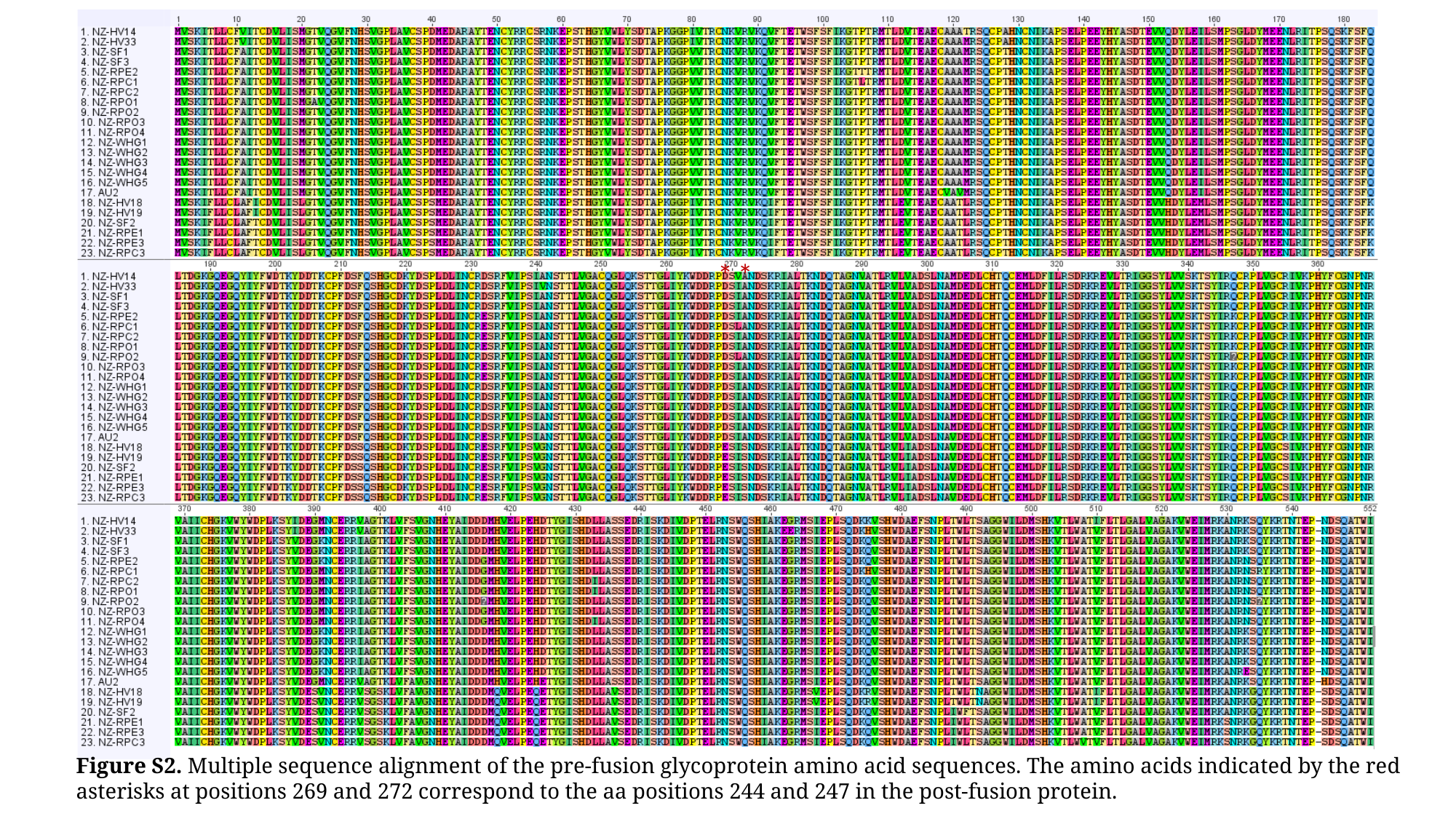

*
*
Figure S2. Multiple sequence alignment of the pre-fusion glycoprotein amino acid sequences. The amino acids indicated by the red asterisks at positions 269 and 272 correspond to the aa positions 244 and 247 in the post-fusion protein.
