# Supplementary material for: Is the Glycoprotein Responsible for the Differences in Dispersal Rates between Lettuce Necrotic Yellows Virus Subgroups?
Source: Viruses. 2022 Jul 20;14(7):1574. doi: 10.3390/v14071574 (PMC9316239; doi:10.3390/v14071574)
Supplement: Supplementary file 1 [file viruses-14-01574-s001.zip › Supplementary Table S1.pdf]

Table S1: Validation using SAVES of predicted 3D structures built by I-TASSER and Robetta. ORF stands for open reading frame

Program	Sample	ERRAT (%)	VERIFY3D (%)	Ramachandran Plot (%)			
				Most Favoured Region	Additional Allowable Region	Generously Allowable Region	Disallowed Region
**I-TASSER**	LNYV-SI AU2	93.4	56.5	66.5	26.2	4.7	2.6
	LNYV-SI HV33 (NZ6)	68.5	36.2	58.2	32.7	6.3	2.8
	LNYV-SII HV19 (NZ1)	76.4	40.8	57.4	33.5	7	2.1
	VSV	92	80.6	78.8	17.3	1.4	2.5
	**Average**	**82.58**	**53.53**				
**ROBETTA**	LNYV-SI AU2	82	90.2	84.5	14.3	0.7	0.5
	LNYV-SI HV33 (NZ6)	86.9	80.1	85.9	13.8	0.2	0
	LNYV-SII HV19 (NZ1)	84.6	88.2	85.9	12.2	0.5	1.5
	LNYV-SII HV19 (NZ1) DIII mutant	85.1	90.4	82.6	15.3	1.4	0.7
	VSV	90.1	87.7	93.9	5.8	0.3	0
	**Average**	**85.74**	**87.32**				
